# Characterization of a Non-Canonical Signal Peptidase Cleavage Site in a Replication Protein from Tomato Ringspot Virus

**DOI:** 10.1371/journal.pone.0162223

**Published:** 2016-09-02

**Authors:** Ting Wei, Joan Chisholm, Hélène Sanfaçon

**Affiliations:** 1 Department of Botany, University of British Columbia, Vancouver, BC, Canada; 2 Summerland Research and Development Centre, Agriculture and Agri-Food Canada, Summerland, BC, Canada; Centro Nacional de Biotecnologia, SPAIN

## Abstract

The NTB-VPg polyprotein from tomato ringspot virus is an integral membrane replication protein associated with endoplasmic reticulum membranes. A signal peptidase (SPase) cleavage was previously detected in the C-terminal region of NTB-VPg downstream of a 14 amino acid (aa)-long hydrophobic region (termed TM2). However, the exact location of the cleavage site was not determined. Using *in vitro* translation assays, we show that the SPase cleavage site is conserved in the NTB-VPg protein from various ToRSV isolates, although the rate of cleavage varies from one isolate to another. Systematic site-directed mutagenesis of the NTB-VPg SPase cleavage sites of two ToRSV isolates allowed the identification of sequences that affect cleavage efficiency. We also present evidence that SPase cleavage in the ToRSV-Rasp2 isolate occurs within a GAAGG sequence likely after the AAG (GAAG/G). Mutation of a downstream MAAV sequence to AAAV resulted in SPase cleavage at both the natural GAAG/G and the mutated AAA/V sequences. Given that there is a distance of seven aa between the two cleavage sites, this indicates that there is flexibility in the positioning of the cleavage sites relative to the inner surface of the membrane and the SPase active site. SPase cleavage sites are typically located 3–7 aa downstream of the hydrophobic region. However, the NTB-VPg GAAG/G cleavage site is located 17 aa downstream of the TM2 hydrophobic region, highlighting unusual features of the NTB-VPg SPase cleavage site. A putative 11 aa-long amphipathic helix was identified immediately downstream of the TM2 region and five aa upstream of the GAAG/G cleavage site. Based on these results, we present an updated topology model in which the hydrophobic and amphipathic domains form a long tilted helix or a bent helix in the membrane lipid bilayer, with the downstream cleavage site(s) oriented parallel to the membrane inner surface.

## Introduction

Signal peptidases (SPases) are ubiquitous membrane-anchored serine proteases that function in bacteria or higher eukaryotes to cleave N-terminal signal sequences of preproteins [1]. In eukaryotes, the endoplasmic reticulum (ER) SPase processes preproteins to release mature soluble proteins into the ER lumen and consequently into the cellular secretory pathway. The ER SPase, as exemplified by the canine microsomal SPase, is a complex of several subunits, each anchored into the lipid bilayer by hydrophobic transmembrane domains [2]. The active sites of the two nearly identical catalytic subunits are located in the ER lumen and recognize cleavage sites that are presented at an optimal distance from the internal surface of the lipid bilayer. Although the primary function of the ER SPase is to release N-terminal signal peptides, it has also been reported to cleave integral membrane proteins at internal or C-terminal sites [3,4].

Cleavage sites recognized by the ER SPase share common features: a cytoplasmic positively charged amino-terminal region (n-region) followed by a hydrophobic α-helix region (h-region) that traverses the membrane lipid bilayer, and a carboxyl-terminal region (c-region) that contains the cleavage site. The typical length of the n-, h- and c-regions are 1–5, 7–15 and 3–7 amino acids (aa), respectively [5]. Helix-breaking residues (Pro or Gly) are often found at the junction between the h- and c-regions and likely allow optimal presentation of the cleavage sites to the catalytic site of the SPase [6,7]. Altering the length of the h-region, the distance of the cleavage sites from the h-region, or structural features within the c-region have been shown to influence the position of the cleavage site and/or cleavage efficiency, sometimes even resulting in the recognition of two or more alternate proximal cleavage sites [7–10]. The cleavage site itself is characterized by a requirement for small and neutral amino acids at the -1 and -3 positions, the so-called -1–3 rule [3,11,12]. In eukaryotic SPase cleavage sites, Ala>Gly,Ser>Thr and Ala,Val>Ser,Thr,Gly are the noted prevalence for the -1 and -3 positions, respectively [13]. Although these common features are conserved in the vast majority of SPase cleavage sites, an alternative membrane topology can also be recognized by the ER SPase, as has been shown for a structural glycoprotein of a pestivirus [14]. This cleavage site was not preceded by a typical hydrophobic α-helix but rather by an amphipathic α-helix that was positioned parallel to the luminal surface of the ER membrane.

Several enveloped positive-strand RNA viruses, notably members of the family *Flaviviridae*, are known to use viral proteases as well as the host ER SPase and signal peptide peptidase to release their membrane-associated structural glycoproteins from larger polyproteins [14–16]. The replication of positive-strand RNA viruses occurs in association with host intracellular membranes, often derived from the ER [17–19]. The formation of membrane-associated viral replication complexes is facilitated by viral integral membrane proteins. In the case of picornaviruses and related viruses, these integral membrane proteins are released from large polyproteins or intermediate polyprotein precursors by the action of viral proteases [20,21]. However, given their association with ER membranes, the possibility that they are also cleaved by membrane-associated host proteases such as the ER SPase cannot be disregarded.

*Tomato ringspot virus* (ToRSV) is a plant picorna-like virus (genus *Nepovirus*, family *Secoviridae*, order *Picornavirales*) [22,23]. The replication complex is anchored to ER membranes by two viral membrane proteins: X2, a protein of unknown function and NTB, a putative helicase with a nucleoside triphosphate-binding motif [24–28]. Both the mature NTB protein and the NTB-VPg polyprotein intermediate have been detected in infected plants [27]. Membrane binding of NTB-VPg is achieved through an N-terminal amphipathic helix and a C-terminal hydrophobic domain that traverses the membrane [28]. Proteinase K treatments indicated that the C-terminal region of NTB as well as the entire VPg is embedded in the ER lumen [27]. This topology was confirmed by the observation that a consensus N-linked glycosylation site (Asn-Xaa-Thr) in the VPg domain is recognized by the ER luminal oligosaccharyltransferase *in vitro* and *in vivo* [24,25]. A putative membrane-associated SPase cleavage was observed when the C-terminal half of the NTB-VPg protein (denoted cNV protein) was expressed *in vitro* in the presence of canine microsomal membranes [24]. However, the exact location of the cleavage site is not known.

In this study, we mapped the SPase cleavage site to a GAAGG sequence using mutational analysis. This cleavage was suboptimal in the wild-type sequence but could be enhanced by mutation of the presumed -1 position to an A (GAAA/G). Other mutations resulted in the recognition of an introduced cleavage site seven amino acid downstream of the natural site. The natural GAAGG cleavage site is located 17 aa downstream of the h-region, a characteristic clearly distinct from the 3–7 aa spacing between the h-region and the cleavage site observed for most SPase cleavage sites. However, a putative amphipathic helix was also predicted immediately downstream of the h-region. Based on these observations, an updated topology model is presented, which implies that a tilted or bent helix is formed in the membrane, resulting in optimal presentation of the SPase cleavage site.

## Materials and Methods

### Computer-based analysis of NTB-VPg

A multiple sequence alignment was performed using the available deduced amino acid sequence of NTB-VPg from different ToRSV isolates [29,30] and the ClustalW2 software. The NTB-VPg sequences of ToRSV isolates can be found under the following GenBank accession numbers: ToRSV-Rasp1 (KM083894, referred to as Rasp1 hereon), ToRSV-Raspberry (NC_003840, referred to as Rasp2 hereon), ToRSV-13C280 (KM083890) and ToRSV-GYV (KM083892). Prediction of SPase cleavage sites was conducted using the SignalP4.1 software [31]. Secondary structure predictions were conducted using the DSC [32], MLRC [33] and PHD [34] software programs and are presented as a consensus of the various prediction programs.

### Plasmid constructions

Plasmids pCITE-cNV, pCITE-cNV (T^610^A) and pCITE-cNV (ΔHR3) are derived from the Rasp2 isolate and were used for *in vitro* translation of the S-cNV-H_6_ protein with in frame fusion of the C-terminal region of NTB-VPg (cNV) to the S-tag at its N-terminus, and a 6xHis tag at its C-terminus. These constructs have been described previously [24]. Please note that the ΔHR3 mutant was previously described under the name ΔTM3 but was renamed ΔHR3 in this manuscript for consistency with the labelling of the HR3 hydrophobic region. pCITE-cNVstop incorporates a stop codon immediately downstream of the VPg domain, eliminating the fusion to the 6xHis tag and allowing the expression of the S-cNV protein. To construct pCITE-cNVstop, a cDNA fragment was amplified using forward primer ToRSV300 (5’-TGCGTTGGATCCGAATTAAGTGCTGAGTTGTTGCTGC-3’, with the inserted restriction site underlined) and reverse primer ToRSV295 (5’-TCTCGGGGATCCTACTGTACAGATTGTGGGCGGAAAACGCGTG-3’), digested with *Bam*HI and inserted into the *Bam*HI site of pCITE-4a (+) (Novagen). Similar pCITE-cNVstop constructs containing the equivalent region derived from the 13C280 and GYV isolates were constructed in the same manner using primer pairs ToRSV295-ToRSV300 and ToRSV345 (5’-TGCGTTGGATCCGAACTTAGTGCCGAGCTCA-3’, forward)-ToRSV346 (5’-TCTCGGGATCCTACTGCACAGACTGAGGCCT-3’, reverse), respectively. To obtain a pCITE-cNVstop construct with the corresponding Rasp1 sequence, primer pair ToRSV302 (5’-TCTCGGAGATCTACTGTACAGATTGCGGCCTGAAAACGCGAG-3’, reverse) -ToRSV307 (5’-TGCGTTAGATCTGAGATGAGTGCTGAGTTATTGCTTAGG-3’, forward) was used and the amplified fragment was digested with *Bgl*II and inserted into the *BamH*I site of pCITE-4a (+). *In vitro* mutagenesis was performed according to the protocol of the QuikChange II-E Site-Directed Mutagenesis kit (Stratagene) using complementary primers that contain the desired mutations. Because mutations were first tested using the S-cNV-H_6_ constructs for Rasp2 and the S-cNV construct for Rasp1, we produced new constructs that incorporated an HA tag upstream of the cNV sequence and the H_6_ tag downstream of the cNV sequence. Selected key mutations incorporated in either the Rasp1 or Rasp2 sequences were retested using these constructs. To construct the HA-cNV-H_6_ plasmids, cDNA fragments corresponding to wild-type or mutated sequences of Rasp1, Rasp2 and GYV cNV protein were synthesized *in vitro* using the GeneArt service of Life Technologies. The fragments were flanked by an *NcoI* site (which contained the AUG start codon immediately prior to the HA tag) at their 5’ end and by a *BamHI* sequence immediately after the stop codon at their 3’ end and were inserted in the corresponding sites of vector pET24D (Novagen). All constructs were verified by sequencing.

### *In vitro* membrane-associated modification assays

*In vitro* coupled transcription and translation assays were performed in the presence or absence of canine pancreatic microsomal membranes (Promega) as previously described [24]. For inhibitor treatments, 1.2 mM SPase inhibitor (MeOSuc-Ala-Ala-Pro-Val chloromethyl ketone, Sigma) was added to the assay. Reactions were run on SDS-polyacrylamide gels (SDS-PAGE). Labeling of proteins with [^35^S] methionine (Perkin Elmer) allowed visualization of the precursor proteins, glycosylated proteins and SPase cleavage products. A phosphorimager (Cyclone Plus, Perkin Elmer) was used to collect the images. Exposure to film was also used to obtain sharper images. Three to six repeats were performed for each mutant with similar results. For each repeat, the corresponding wild-type Rasp2 and/or Rasp1 sequences were included as a comparison.

## Results

### Sequence alignment of NTB-VPg from different ToRSV isolates

**Fig 1:**
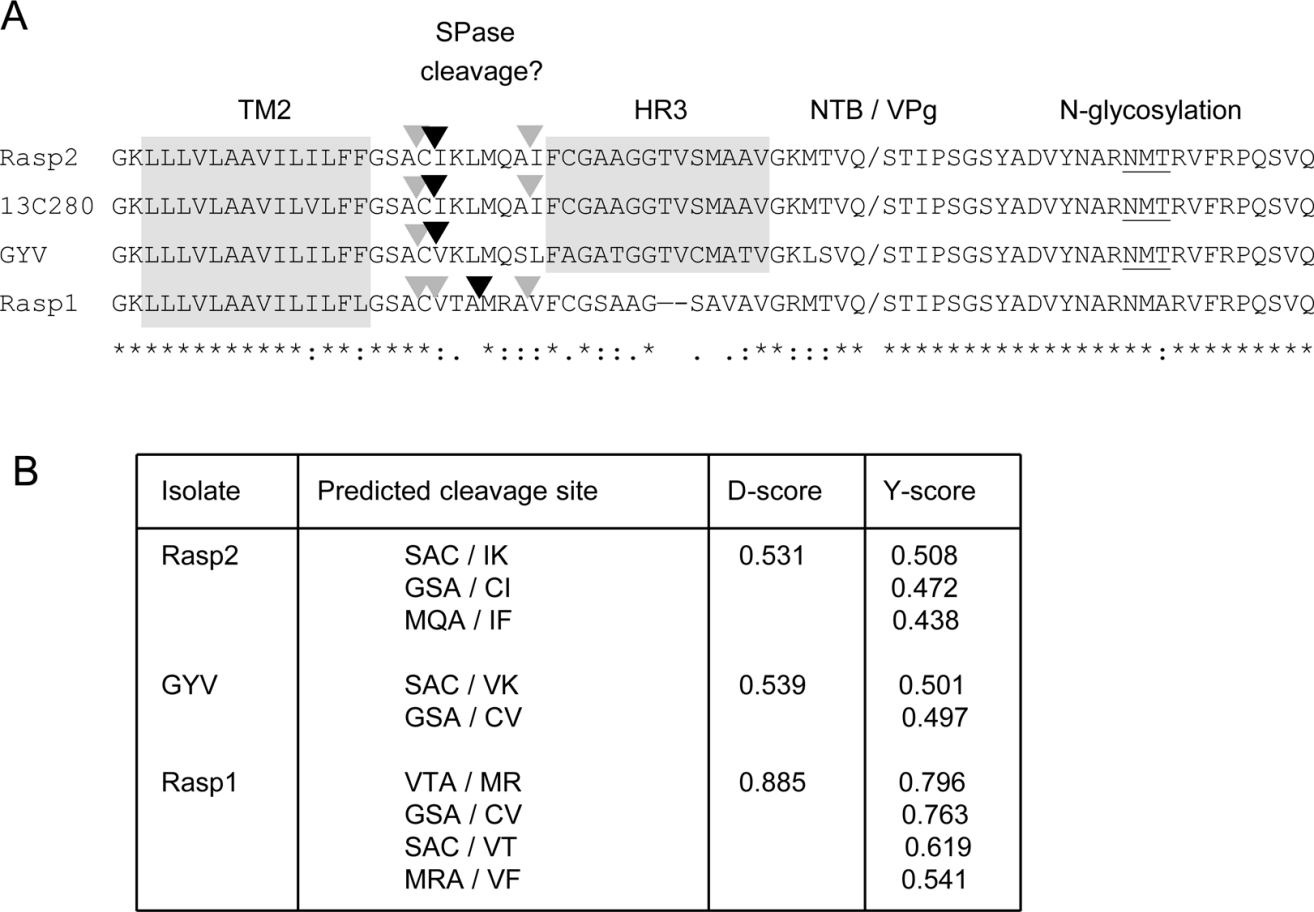
Prediction of SPase cleavage sites in the C-terminal region of NTB-VPg from various ToRSV isolates. (A) Amino acid alignment of the C-terminal region of NTB-VPg from different ToRSV isolates. Previously identified motifs are highlighted with the grey boxes and defined above the alignment and in the text. The border between the NTB and VPg domain (NTB/VPg) is also shown. As defined in ClustalW2, an asterisk (*) indicates conserved residues, a colon (:) indicates residues with strongly similar properties and a period (.) indicates residues with weakly similar properties. SPase cleavages predicted by the SignalP 4.1 software are shown by the arrowheads above each sequence, with the black arrowhead representing the most strongly predicted cleavage site. (B) Prediction of SPase cleavage sites using the SignalP 4.1 software. For the Y-score, only scores above 0.4 are shown.

Amino acid alignment of the NTB-VPg sequences revealed that the two main membrane association domains previously identified in the ToRSV Rasp2 isolate were conserved in other ToRSV isolates (S1 Fig). The N-terminal putative amphipathic helix was identical amongst all isolates and the highly hydrophobic C-terminal transmembrane domain (TM2) was highly conserved, with the exception of an I^558^ to V substitution (numbering from the N-terminus of the NTB domain) in 13C280 and an F^561^ to L substitution in Rasp1 (S1 Fig, Fig 1A). These mutations are not predicted to change the overall hydrophobicity or membrane topology of the TM2 domain. In contrast, the region of NTB downstream of the TM2 domain exhibited remarkable differences in Rasp1 compared to the other isolates (Fig 1A). These differences included the region around the previously predicted MQA/I SPase cleavage site [24] and the downstream mildly hydrophobic HR3 region. In addition to point mutations, deletion of two aa (TV^580-581^) in the HR3 domain of Rasp1 decreased its overall hydrophobicity (Fig 1A). Although the VPg domain was highly conserved amongst all isolates, the N-linked glycosylation site (NMT^610^) in the VPg region was absent in Rasp1 and was replaced by the NMA^608^ sequence. In summary, a number of amino acid substitutions were observed in the C-terminal region of ToRSV-Rasp1 NTB-VPg, which may alter its conformation in the membrane and consequently its membrane-associated modifications.

We used the updated SignalP4.1 software [31] to re-evaluate potential SPase cleavage sites in the NTB-VPg sequences. Because the software is designed to recognize N-terminal signal peptides, we input truncated sequences that contain between one and five aa upstream of the TM2 domain. As results were essentially similar within this range, we present results obtained using one aa upstream of the TM2 domain. SPase cleavage was predicted for all ToRSV isolates with varying degrees of confidence (Fig 1B, see D-score; a D-score of 0.5 or above is considered the threshold for SPase cleavage). The D-score is only provided for the most likely cleavage sites. However, additional possible cleavage sites can be inferred from the Y-score allotted for each residue (Fig 1B). The positions of the predicted cleavage sites are shown above the sequence (Fig 1A). For Rasp2, 13C280 and GYV, the most likely cleavage sites were predicted to be 3–4 aa downstream of the TM2 domain. A helix-breaking Gly immediately downstream of TM2 would be present at the -3 or -4 position of these predicted cleavage sites. The previously predicted MQA-IF cleavage site [24] had a slightly lower Y-score and cleavage at this position was not predicted for GYV. Results were similar for Rasp1, although an additional cleavage site was predicted and the degree of confidence in SPase cleavage was higher than with the other isolates.

### Differences in SPase cleavage efficiency in the C-terminal region of NTB-VPg amongst ToRSV isolates

**Fig 2:**
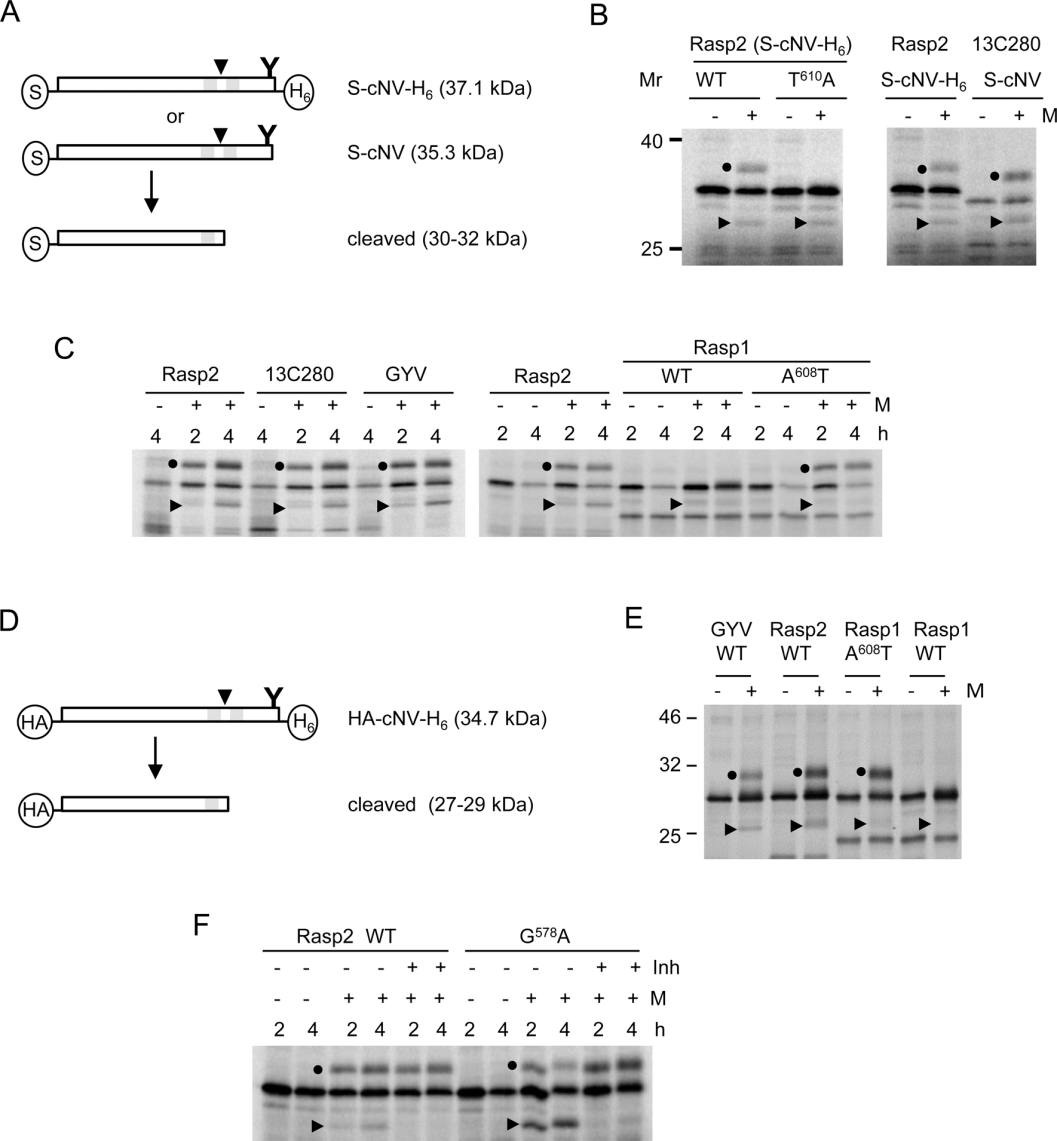
Analysis of SPase cleavage and glycosylation in the C-terminal region of the NTB-VPg protein of various ToRSV isolates using *in vitro* membrane associated-modification assays. **(**A) Schematic representation of the cNV constructs with the predicted size of the unmodified precursor protein and SPase cleaved product. The arrow indicates the predicted SPase cleavage site and Y represents the predicted glycosylation site. The S-tag or 6xHis in frame fusions are shown in the circles. (B) Wild-type (WT) or T^610^A mutant versions of the S-cNV-H_6_ protein derived from the Rasp2 isolate and the WT S-cNV protein derived from the 13C280 isolate were expressed *in vitro* in the presence (+) or absence (-) of canine microsomal membranes (M) as indicated above each lane. Reactions were performed at room temperature for six hours. Translation products and membrane-associated modified products were separated by 11% SDS-PAGE. The glycosylated forms of cNV are indicated with black dots and the SPase cleavage products are shown with black triangles. The migration of molecular mass markers (Mr) is indicated in kDa. (C) WT S-cNV protein derived from the Rasp2, 13C280, GYV or Rasp1 sequences were tested using *in vitro* membrane-associated modification assays as in (B). A mutated (A^608^T) version of the Rasp1 sequence was also tested. Reactions were conducted for 2 or 4 hours (h) as indicated. (D) Schematic representation of the HA-cNV-H_6_ constructs. The calculated sizes of the unmodified precursor protein and of the SPase cleaved product are indicated. (E) *In vitro* membrane-associated modification assays of HA-cNV-H_6_ constructs. Reactions were conducted for 4.5 h. (F) *In vitro* membrane-associated modification assays were conducted in the presence or absence of an SPase inhibitor (MeOSuc-Ala-Ala-Pro-Val chloromethyl ketone, Sigma) at a final concentration of 1.2 mM. WT or mutated (G^578^A, see below) S-cNV-H_6_ proteins derived from the Rasp2 sequence were tested.

To test for membrane-associated modifications of the NTB-VPg proteins, we used *in vitro* translation assays in the presence of canine microsomal membranes, a commercial preparation of ER-derived membranes [24]. Glycosylation at the NMT sequence in the Rasp2 VPg domain was previously demonstrated using either the full-length NTB-VPg polyprotein or N-terminally truncated NTB-VPg polyproteins of various lengths [24]. These truncated proteins included cNV which contains the C-terminal 254 aa of the NTB domain and the entire VPg domain (Fig 2A, S1 Fig). This previous observation confirmed that the membrane topology of the C-terminal region of the full-length or truncated NTB-VPg was similar. In both cases, the VPg domain was translocated in the ER lumen and thereby, accessible to the oligosaccharyl transferase. Putative SPase cleavage was previously observed for the truncated proteins but was not easily detectable in the 69 kDa full-length NTB-VPg due to the presence of multiple background bands that migrated in the 62–65 kDa range in SDS-polyacrylamide gels [24]. These bands coincided with the migration of the expected SPase cleavage product. This was likely due to translation initiation at internal AUGs or premature translation termination. We therefore continued the analysis of SPase cleavage using the truncated cNV protein.

The previously described ToRSV-Rasp2 cNV protein (S-cNV-H_6_) is fused in frame with an S-tag at its N-terminus and a 6xHis tag at its C-terminus. As previously shown [24], this protein was glycosylated and cleaved in the presence of membranes (Fig 2B). The membrane concentration was titrated to maximize membrane modification efficiency but avoid inhibitory effects on translation efficiency. It is noted that under these conditions the S-cNV-H_6_ protein was only partially glycosylated, possibly due to limiting amounts of membranes. Consistent with previous observations [24], mutation of the N-glycosylation site (T^610^A) in the VPg domain prevented the production of the glycosylated protein but did not prevent the release of the cleavage product (Fig 2B). To ensure that the C-terminal fusion to the 6xHis tag (which also included several aa from the pCITE4a vector) did not alter the membrane topology of the protein, we produced derivatives of S-cNV-H_6_ by inserting a stop codon immediately downstream of the VPg domain to reconstitute the natural C-terminal end of the NTB-VPg polyprotein (S-cNV) (Fig 2A). As expected, the unmodified S-cNV protein migrated slightly faster than the S-cNV-H_6_ protein, but was also glycosylated and cleaved (Fig 2B and 2C).

As mentioned above, Rasp2 and 13C280 are nearly identical in the cNV region. Not surprisingly, membrane-associated modifications of the Rasp2 and 13C280 S-cNV proteins were similar (Fig 2C). Although showing more sequence variation, the S-cNV protein derived from the GYV isolate was also glycosylated and cleaved in the presence of membranes. As previously observed [24], accumulation of the cleaved product increased over time indicating that it is a post-translational event at least *in vitro*. In contrast, N-glycosylation occurred co-translationally.

The Rasp1 sequence does not include a glycosylation site in the VPg domain and consequently was not glycosylated (Fig 2C). However, glycosylation was observed in an A^608^T mutant that restored the NMT glycosylation site in the VPg domain (Fig 2C). This suggests that the Rasp1 S-cNV protein adopts a topology similar to that observed for the other isolates, with the VPg translocated into the lumen of the membrane. Cleavage products were not easily detectable for Rasp1 S-cNV, using either the wild-type sequence or the A^608^T mutant derivative but the detection may have been obscured by the presence of background bands close to the expected migration position of the cleavage product. Indeed, SPase cleavage of the Rasp1 HA-cNV-H_6_ (wild-type or A^608^T mutant) protein in which the S-tag was replaced by an HA tag, was detected, but was inefficient compared to the corresponding Rasp2 or GYV constructs (Fig 2D and 2E).

To further confirm that the cleaved products were released by SPase cleavage, a previously described SPase inhibitor (MeOSuc-Ala-Ala-Pro-Val chloromethyl ketone) [14] was added to the *in vitro* assay. The wild-type ToRSV-Rasp2 S-cNV-H_6_ protein and a mutant (G^578^A) with increased SPase cleavage (see below) were used as substrates. Addition of the inhibitor reduced the accumulation of the cleaved product (Fig 2F). Taken together, the results indicate that the cNV proteins of all ToRSV isolates adopt similar membrane topology and that they are cleaved by the SPase, although with varying efficiency.

### Complete or partial deletion of a weak hydrophobic domain (HR3) reduces the efficiency of SPase cleavage

**Fig 3:**
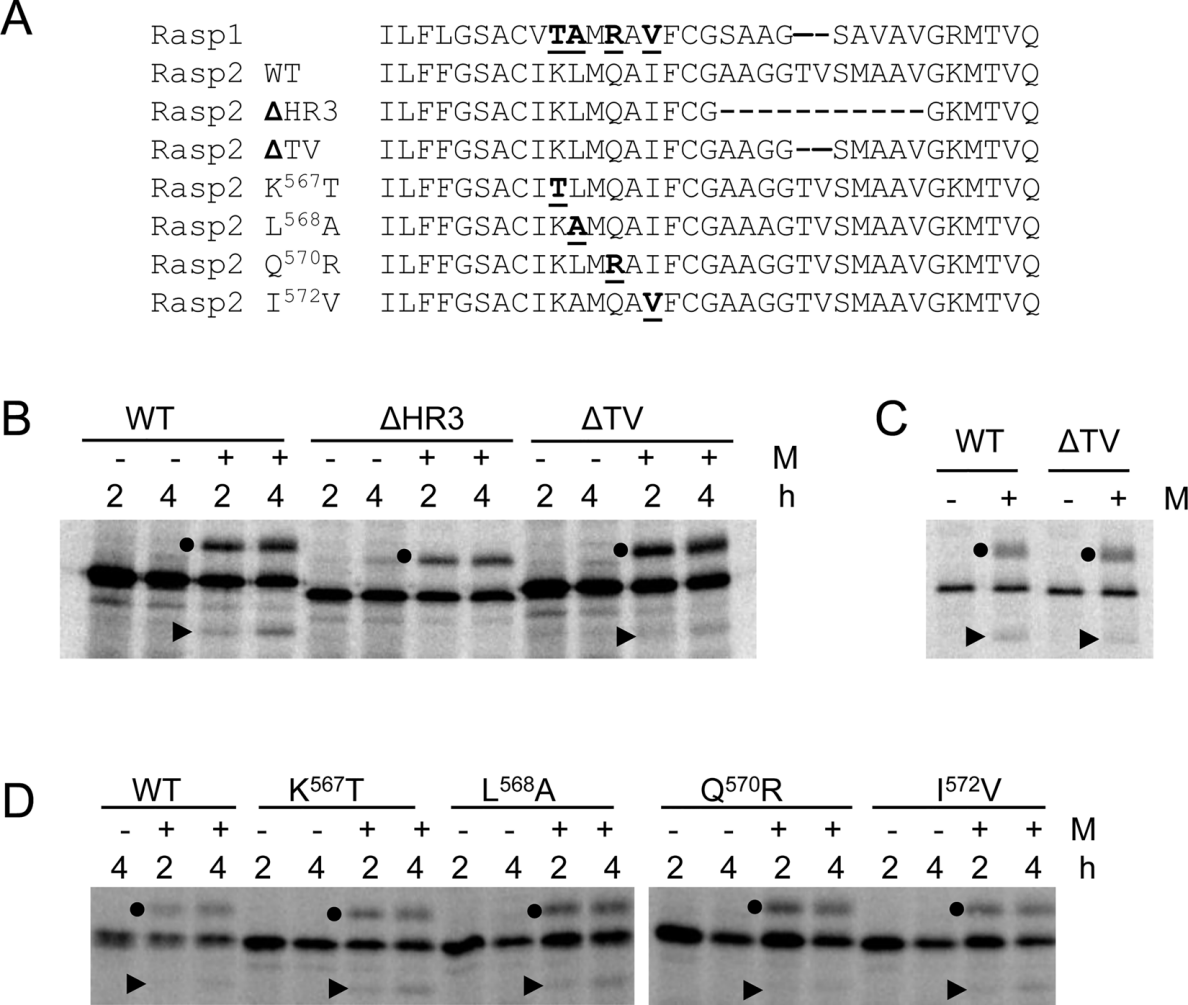
*In vitro* membrane-associated modification assays of wild-type or mutated versions of Rasp2 S-cNV-H_6_. **(**A) Amino acid (aa) alignment of the C-terminal region of the NTB domain for relevant mutants of Rasp2. The Rasp1 sequence is also shown for reference. Mutated aa are underlined. The alignment starts with the four C-terminal aa of the TM2 domain and ends with the C-terminal aa of the NTB domain. (B and D) *In vitro* membrane-associated modification assays of WT or mutant derivatives of Rasp2 S-cNV-H_6_ were conducted as in Fig 2C. (C) *In vitro* membrane-associated modification assays of the WT or ΔTV mutant derivative of Rasp2 HA-cNV-H_6_. Reactions were conducted for 4h.

As mentioned above, ToRSV-Rasp1 differs from the other isolates in the region downstream of the TM2 domain, including the deletion of two aa (TV) in the HR3 domain (Figs 1A and 3A). As previously shown [24], a Rasp2 S-cNV-H_6_ mutant with a large deletion of HR3 (Rasp2ΔHR3) is apparently not cleaved by the SPase although it is still glycosylated (Fig 3B). In addition, deletion of the TV sequence (ΔTV) in either the Rasp2 S-cNV-H_6_ or HA-cNV-H_6_ constructs resulted in a slight but reproducible reduction in the efficiency of SPase cleavage (Fig 3B and 3C). These results strongly indicate that the HR3 region plays an important role in the SPase processing.

### Single point mutations in the region immediately upstream of the HR3 domain that correspond to differences between the Rasp1 and Rasp2 isolates only modestly influence the efficiency of SPase cleavage

We tested the impact of individual aa substitutions corresponding to differences observed between Rasp2 and Rasp1 cNV proteins in the region immediately upstream of HR3, including K^567^T, L^568^A, Q^570^R and I^572^V mutations (Fig 3A). The K^567^T, L^568^A and I^572^V mutations introduced into the Rasp2 S-cNV-H_6_ sequence did not significantly influence the efficiency of SPase cleavage while the Q^570^R mutation slightly reduced but did not eliminate SPase cleavage (Fig 3D). In conclusion, single mutations introduced into the region immediately upstream of HR3 had only modest effects on the efficiency of SPase cleavage. This result does not exclude the possibility that double or triple mutations in this region could have a greater impact.

### Point mutations in the HR3 region affect SPase cleavage efficiency and provide evidence that SPase cleavage occurs within a GAAGG sequence in the wild-type ToRSV-Rasp2 sequence

**Fig 4:**
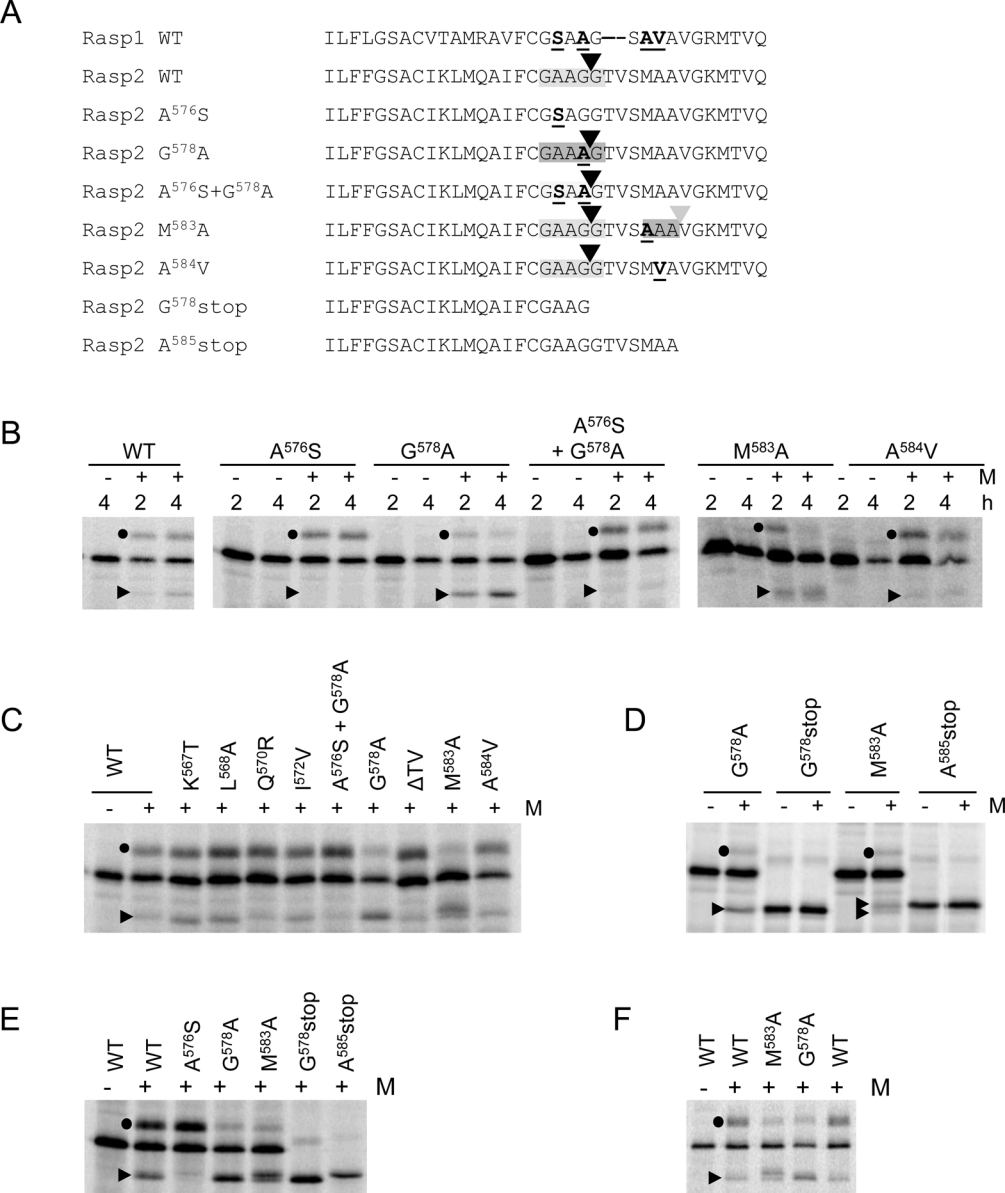
Mapping of SPase cleavage sites in wild-type or mutated versions of Rasp2 S-cNV-H_6_. **(**A) Amino acid alignment of the C-terminal region of the NTB domain of relevant mutants of Rasp2 (as in Fig 3A). Mutated aa are underlined. In the case of the G^578^stop and A^585^stop mutants, stop codons were introduced at the indicated position resulting in the production of a truncated protein. The approximate deduced location of the SPase cleavage sites are shown with grey shadings, with darker shading corresponding to higher efficiency of SPase cleavage. The black arrow indicates the deduced position of the natural SPase cleavage site in the WT Rasp2 sequence while the grey arrow indicates the position of the additional SPase cleavage site detected in the M^583^A mutant. (B) *In vitro* membrane-associated modification assays of WT or mutant derivatives of Rasp2 S-cNV-H_6_ were conducted as in Fig 2C. (C and D) Experiments were conducted as in B, but reactions were allowed to proceed for four hours. (E and F) Experiments were conducted as in C, but using WT or mutant derivatives of Rasp2 HA-cNV-H_6_.

We introduced single and double amino acid mutations in the S-cNV-H_6_ Rasp2 protein at positions 575–579 within the HR3 region. The Rasp2 GAAGG^579^ sequence differs by two amino acids from the Rasp1 GSAAG^579^ sequence (Fig 4A). Increased SPase processing was observed for the G^578^A mutation that creates the sequence GAAAG (Fig 4B). In contrast, cleavage was reduced after mutation of A^576^ to S (creating the sequence GSAGG). A double mutant (A^576^S+G^578^A) that reconstituted the Rasp1 sequence (GSAAG) was cleaved although at reduced efficiency compared to the wild-type Rasp2 sequence. Considering the reported preference for Ala over Ser or Gly at the -3 and -1 positions of eukaryotic SPase cleavage sites [13], the increased cleavage observed for the G^578^A mutation was likely due to cleavage after the introduced AAA sequence (i.e. GAAA/G with the cleavage site indicated with the slash). To provide further evidence for this suggestion, we introduced a stop codon in the wild-type Rasp2 sequence to produce a truncated protein with the last amino acid being G^578^. As anticipated, this mutant (G^578^stop) co-migrated with the cleavage product obtained after SPase processing of the G^578^A mutant (Fig 4D). Similar results were obtained when mutations were introduced into the HA-cNV-H_6_ Rasp2 protein (Fig 4E). Based on the similar migration of the cleaved products released after SPase cleavage of the WT sequence or G^578^A mutant on SDS-PAGE (Fig 4C, 4E and 4F), it is likely that cleavage occurred within the GAAGG sequence in the wild-type Rasp2 cNV construct. This sequence includes three possible SPase cleavage sites, each with either an A or a G at the -1 and -3 positions (Table 1). The G^578^A mutation, which enhanced SPase cleavage, improves the context of only one of the three cleavage site (CS1 in Table 1) by inserting a preferred Ala at the -1 position. This mutation is not predicted to affect the context of the two alternative cleavage sites (Table 1). In addition, the A^576^S mutation, which drastically reduced SPase cleavage, introduces a sub-optimal Ser in the -3 position of the CS1 cleavage site but would not be predicted to affect the context of the two alternate cleavage sites (Table 1). Based on these results, we tentatively propose that cleavage occurred at the CS1 cleavage site (GAAG^578^/G) in the Rasp2 wild-type sequence.

**Table 1:** Analysis of the context of three possible SPase cleavage sites in wild-type or mutated Rasp2 cNV constructs.

Construct name ^a^	Sequence ^b^	Context (-1,-3 rule)
WT (CS1)	FCGAAG **/ **GTV	-1G -3A
WT (CS2)	FCGAA **/ **GGTV	-1A -3G
WT (CS3)	FCGAAGG **/ **TV	-1G -3A
G^578^A (CS1)	FCGAA**A** **/ **GTV	-1A -3A (better context than WT)
G^578^A (CS2)	FCGAA **/** **A**GTV	-1A -3G (unchanged)
G^578^A (CS3)	FCGAA**A**G **/ **TV	-1G -3A (unchanged)
A^576^S (CS1)	FCG**S**AG **/ **GTV	-1G -3S (worse context than WT)
A^576^S (CS2)	FCG**S**A **/ **GGTV	-1A -3G (unchanged)
A^576^S (CS3)	FCG**S**AGG **/ **TV	-1G -3A (unchanged)
A^576^S + G^578^A (CS1)	FCG**S**A**A** **/ **GTV	-1A -3S (worse than G^578^A, better than A^576^S)
A^576^S + G^578^A (CS2)	FCG**S**A **/** **A**GTV	-1A -3G (unchanged)
A^576^S + G^578^A (CS3)	FCG**S**A**A**G **/ **TV	-1G -3A (unchanged)

CS, cleavage site

^a^ Three possible cleavage sites are shown for each construct (CS1 to CS3)

^b^ Bold underlined letters show the sites of mutations

We also introduced mutations in positions 583–585 that differed in the two ToRSV isolates (MAA for Rasp2 and AVA for Rasp1) (Fig 4A). An A^584^V mutation introduced into the Rasp2 S-cNV-H_6_ sequence did not have a significant effect on the efficiency of SPase processing (Fig 4B and 4C). In contrast, SPase cleavage was enhanced in the M^583^A mutant, which created an AAA sequence, when this mutation was introduced in either the Rasp2 S-cNV-H_6_ or HA-cNV-H_6_ constructs (Fig 4B–4F). Interestingly, two cleavage products were detected for this mutant (Fig 4C–4F). The smaller product co-migrated with those derived from the wild-type sequence or from the G^578^A mutant and was likely the result of cleavage at the proposed GAAG^578^/G cleavage site (Fig 4F). The larger product was present in higher concentration and likely resulted from cleavage after the newly introduced AAA^585^ sequence (Fig 4A) as it co-migrated with a truncated protein produced by introduction of a stop codon after the codon for A^585^ (A^585^stop mutant) (Fig 4D and 4E). This result strongly suggests that SPase cleavage occurred at two positions separated by seven aa in the M^583^A mutant, with cleavage after the optimal AAA^585^ sequence being favored.

### Mutations of ToRSV-Rasp1 cNTB-VPg enhance signal peptidase cleavage

**Fig 5:**
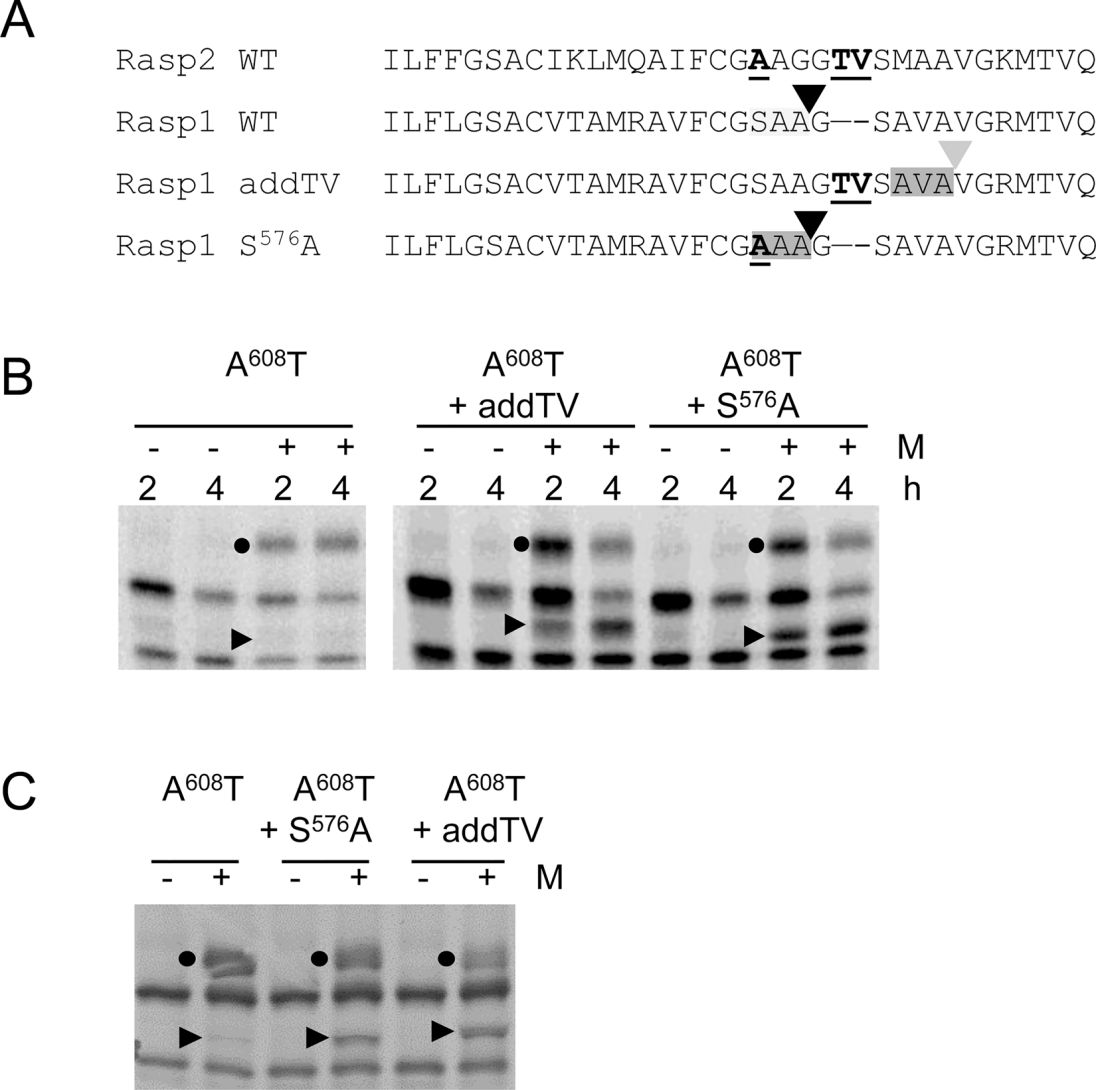
*In vitro* translation of Rasp1 S-cNV and mutant derivatives. **(**A) Amino acid alignment of the C-terminal region of the NTB domain of relevant mutants of Rasp1. Mutated aa are underlined. The Rasp2 sequence is shown at the top of the alignment for reference. The approximate deduced location of the SPase cleavage sites are shown with grey shadings. (B) *In vitro* membrane-associated modification assays of WT or mutant derivatives of the Rasp1 A^608^T S-cNV construct were conducted as in Fig 2C. (C) Experiments were conducted as in B with the exceptions that the mutations were inserted in the Rasp1 A^608^T HA-cNV-H_6_ construct and reactions were incubated for 4.5 h in the presence of microsomal membranes.

Key mutations were also introduced in the Rasp1 A^608^T mutant to mimic the Rasp2 sequence (Fig 5A). As described above, the A^608^T mutation recreates the VPg N-glycosylation site, allowing the monitoring of membrane insertion and SPase cleavage simultaneously. Glycosylation was detected for all Rasp1 cNV mutants, using either the S-cNV constructs (Fig 5B) or HA-cNV-H_6_ constructs (Fig 5C), confirming translocation of the VPg into the membrane lumen. The S^576^A mutation, which creates a favorable AAA sequence at the position corresponding to the proposed GAAG^578^/G cleavage site in the wild-type Rasp2 sequence, was efficiently cleaved by the SPase (Fig 5B and 5C). Introduction of the two missing amino acids in the HR3 domain (addTV mutant) to Rasp1 A^608^T also resulted in detectable SPase cleavage (Fig 5B and 5C). However, the size of the cleaved protein was slightly larger, suggesting that cleavage occurred after the downstream AVA^583^ sequence, which includes an optimal Ala at the -1 and -3 positions (Fig 5A). It is interesting to note that the AVA sequence was not efficiently recognized as an SPase cleavage site in the absence of the TV residues, suggesting that other factors, possibly the conformation of the protein in the membrane, also influence SPase cleavage.

## Discussion

In this study, we describe an SPase cleavage site located in the C-terminal region of an ER-associated replication protein from a plant picorna-like virus. Canonical signal peptides are normally located in the N-terminal region of secreted proteins [35]. However, the ER SPase is also able to cleave at sites located in the C-terminal region of proteins [36]. The ToRSV NTB-VPg SPase cleavage site shares common features with typical SPase cleavage sites, including a positively charged amino acid (lysine) upstream of the transmembrane domain, a core hydrophobic region rich in leucines and small amino acids at the -3 and -1 positions of the cleavage site. However, one feature of the ToRSV NTB-VPg SPase cleavage site is atypical. Indeed, the distance between the end of the TM2 hydrophobic domain and the mapped cleavage site (approximately 17 residues) exceeds that known for regular signal peptides (3–7 residues) [35].

The ER SPase is a membrane protein complex. The catalytic subunits have a transmembrane domain upstream of the active site that is translocated into the membrane lumen [1]. This topology forces specific positioning of the enzyme active site in relation to the lipid bilayer. As a consequence, SPase cleavage sites must be correctly presented to the active site of the enzyme which is close to the surface of the membrane [10]. It has been shown that the optimal distance between the end of the hydrophobic region and the cleavage site is 4–5 residues [37]. Thus, it is not clear how the ToRSV NTB-VPg cleavage site is exposed to and recognized by the active site of the SPase.

**Fig 6:**
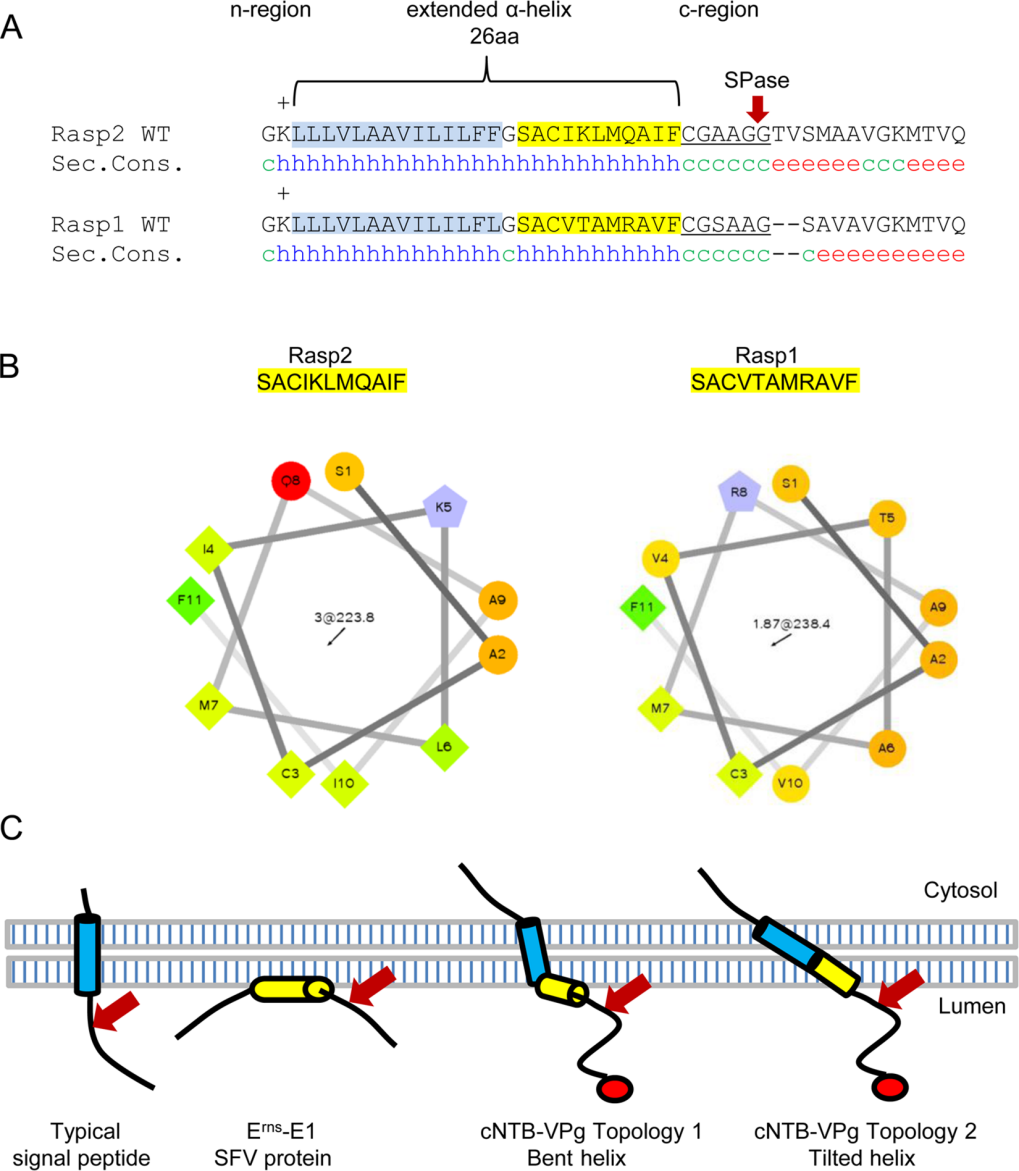
Membrane topology model for the ToRSV NTB-VPg protein. (A). Secondary structure prediction of the C-terminal region of the Rasp1 and Rasp2 NTB domain. The predicted n-region includes a positively charged (+) amino acid residue K. A 26 aa-long α-helix is predicted immediately downstream, with the N-terminal region consisting of the previously identified hydrophobic TM2 transmembrane domain (highlighted in blue) and the C-terminal region having amphipathic properties (highlighted in yellow). The c-region (underlined) and the most likely cleavage site (indicated with the red arrow) are shown. Secondary structure of this region was predicted as described in Materials and Methods and the consensus secondary structures are shown with h, c and e representing α-helices, random coils and extended strands, respectively. (B) Helical projections of the putative amphipathic helices. Amino acid residues used for helical projections are indicated on the top. The hydrophilic residues are shown as circles or hexagons, and hydrophobic residues are shown as diamonds. (C) Membrane topology models for the NTB-VPg protein in comparison to other characterized SPase cleavage sites. The topology of a typical signal peptide is shown on the left with the SPase processing site depicted by the red arrow. The topology of the SPase cleavage in the E^rns^-E1 SFV protein is also shown. For the ToRSV cNTB-VPg substrate, two possible membrane topologies are shown. In Topology 1, the TM2 hydrophobic domain (blue) traverses the membrane and a kink in the α-helix allows the downstream amphipathic helix (yellow) to lay parallel to the surface of the membrane with its hydrophilic side on the luminal face of the membrane. In Topology 2, the 26 aa-long α-helix forms a long tilted structure traversing the membrane.

Secondary structure prediction softwares (DSC, MLRC and PHD) imply the existence of a long α-helix in the Rasp2 and Rasp1 NTB-VPg sequences that encompasses not only the hydrophobic TM2 domain (highlighted in blue in Fig 6A) but also the 12 amino acids further downstream. Projection of the downstream region of the predicted long helix (in yellow) reveals its amphipathic helix property (Fig 6B), which is predicted for both Rasp2 and Rasp1 NTB-VPg sequences, although with a stronger degree of confidence for Rasp2. Therefore, it is possible that the ToRSV NTB-VPg protein adopts an unusual topology, with the hydrophobic region of the predicted helix, which is 26 aa in length, traversing the membrane and the amphipathic region oriented parallel to the luminal side of the membrane (Topology 1, Fig 6C). A bend in the transmembrane domain, possibly facilitated by the glycine residue (between the blue and yellow boxes), would be necessary to allow this topology to occur [38]. By adopting this topology, the cleavage site, which is located downstream of the putative amphipathic helix, is brought closer to the active site of the signal peptidase for processing. Alternatively, it is possible that the 26 aa-long helix forms a single transmembrane domain (Topology 2, Fig 6C). In this case, the helix may be tilted to allow the entire region to be buried in the lipid membrane. Tilting of long hydrophobic regions to accommodate the width of the membrane has been observed in some membrane proteins [38]. The recognition of two alternative cleavage sites separated by seven aa in the M^583^A mutant would also be explained by the two proposed topologies, as the region immediately downstream of the 26 aa long helix would also be oriented parallel to the membrane (Fig 6C). Signal peptidase cleavage after an amphipathic helix was previously observed in classic swine fever virus E^rns^-E1 protein [14], demonstrating that the eukaryotic ER SPase enzyme can accommodate alternative substrate membrane topologies as long as the cleavage site is properly exposed to its catalytic site. The topological models presented above suggest that the ToRSV NTB-VPg protein may represent yet another type of non-canonical SPase substrates.

The biological function of the sub-optimal SPase cleavage of the ToRSV NTB-VPg protein remains to be investigated in the context of the virus infection cycle. The proposed GAAG^578^/G SPase cleavage site is 14 aa upstream of the natural NTB-VPg cleavage site, which is recognized by the viral protease (Fig 1A). Thus, SPase cleavage of the NTB-VPg protein would release an alternate form of the VPg in the lumen of the ER membranes. However, it is unlikely that this alternate luminal VPg would play an active role in virus replication, a process that occurs on the cytoplasmic face of the ER membranes. Indeed, we have previously showed that the ToRSV VPg-Pro-Pol’ polyprotein, a cytoplasmic soluble polyprotein, is peripherally associated with ER membranes active in virus replication and have suggested that this polyprotein may act as a donor for a replication-competent VPg [39]. Alternatively, SPase cleavage could regulate the membrane topology of NTB-VPg. Oligomers of the ToRSV NTB-VPg are detected in ER membranes-enriched fractions [25] and oligomerization was proposed to partially depend on the luminal HR3 hydrophobic domain [28]. SPase cleavage within this domain would weaken these intermolecular interactions and could alter the architecture and/or activity of the replication complex. Testing these hypotheses will require introduction of mutations that enhance or decrease the SPase cleavage (e.g., the G^578^A and A^576^S mutations described in this study) in infectious ToRSV cDNA clones, once they become available.

## Supporting Information

S1 Fig Amino acid alignment of NTB-VPg from different ToRSV isolates. Starting amino acid for the cNV truncated protein is indicated with an arrow. Previously identified motifs are highlighted with the grey boxes and defined above the alignment and in the text. The border between the NTB and VPg domain (NTB/VPg) is also shown. As defined in ClustalW2, an asterisk (*) indicates conserved residues, a colon (:) indicates residues with strongly similar properties and a period (.) indicates residues with weakly similar properties.
(TIF)

## References

[1] Paetzel M, Karla A, Strynadka NC, Dalbey RE. Signal peptidases. Chem Rev. 2002;102: 4549–4580. 12475201 10.1021/cr010166y

[2] Shelness GS, Lin L, Nicchitta CV. Membrane topology and biogenesis of eukaryotic signal peptidase. J Biol Chem. 1993;268: 5201–5208. 8444896

[3] Nilsson I, Johnson AE, von Heijne G. Cleavage of a tail-anchored protein by signal peptidase. FEBS Lett. 2002;516: 106–108. 11959113 10.1016/s0014-5793(02)02511-5

[4] Robakis T, Bak B, Lin SH, Bernard DJ, Scheiffele P. An internal signal sequence directs intramembrane proteolysis of a cellular immunoglobulin domain protein. J Biol Chem. 2008;283: 36369–36376. 10.1074/jbc.M807527200 18981173 PMC2662301

[5] Auclair SM, Bhanu MK, Kendall DA. Signal peptidase I: cleaving the way to mature proteins. Protein Sci. 2012;21: 13–25. 10.1002/pro.757 22031009 PMC3323777

[6] Perlman D, Halvorson HO. A putative signal peptidase recognition site and sequence in eukaryotic and prokaryotic signal peptides. J Mol Biol. 1983;167: 391–409. 6345794 10.1016/s0022-2836(83)80341-6

[7] Nothwehr SF, Gordon JI. Eukaryotic signal peptide structure/function relationships. Identification of conformational features which influence the site and efficiency of co-translational proteolytic processing by site-directed mutagenesis of human pre(delta pro)apolipoprotein A-II. J Biol Chem. 1989;264: 3979–3987. 2537299

[8] Nothwehr SF, Gordon JI. Structural features in the NH2-terminal region of a model eukaryotic signal peptide influence the site of its cleavage by signal peptidase. J Biol Chem. 1990;265: 17202–17208. 2120214

[9] Nothwehr SF, Hoeltzli SD, Allen KL, Lively MO, Gordon JI. Residues flanking the COOH-terminal C-region of a model eukaryotic signal peptide influence the site of its cleavage by signal peptidase and the extent of coupling of its co-translational translocation and proteolytic processing in vitro. J Biol Chem. 1990;265: 21797–21803. 2123875

[10] Nilsson I, Whitley P, von Heijne G. The COOH-terminal ends of internal signal and signal-anchor sequences are positioned differently in the ER translocase. J Cell Biol. 1994;126: 1127–1132. 8063852 10.1083/jcb.126.5.1127 PMC2120157

[11] von Heijne G. Patterns of amino acids near signal-sequence cleavage sites. Eur J Biochem. 1983;133: 17–21. 6852022 10.1111/j.1432-1033.1983.tb07424.x

[12] Folz RJ, Nothwehr SF, Gordon JI. Substrate specificity of eukaryotic signal peptidase. Site-saturation mutagenesis at position -1 regulates cleavage between multiple sites in human pre (delta pro) apolipoprotein A-II. J Biol Chem. 1988;263: 2070–2078. 3276681

[13] Choo KH, Ranganathan S. Flanking signal and mature peptide residues influence signal peptide cleavage. BMC Bioinformatics. 2008;9 Suppl 12: S15 10.1186/1471-2105-9-S12-S15 19091014 PMC2638155

[14] Bintintan I, Meyers G. A new type of signal peptidase cleavage site identified in an RNA virus polyprotein. J Biol Chem. 2010;285: 8572–8584. 10.1074/jbc.M109.083394 20093364 PMC2838279

[15] Moradpour D, Penin F. Hepatitis C virus proteins: from structure to function. Curr Top Microbiol Immunol. 2013;369: 113–142. 10.1007/978-3-642-27340-7_5 23463199

[16] Pene V, Hernandez C, Vauloup-Fellous C, Garaud-Aunis J, Rosenberg AR. Sequential processing of hepatitis C virus core protein by host cell signal peptidase and signal peptide peptidase: a reassessment. J Viral Hepat. 2009;16: 705–715. 10.1111/j.1365-2893.2009.01118.x 19281487

[17] Laliberte JF, Sanfacon H. Cellular remodeling during plant virus infection. Annu Rev Phytopathol. 2010;48: 69–91. 10.1146/annurev-phyto-073009-114239 20337516

[18] Sanfacon H. Replication of positive-strand RNA viruses in plants: Contact points between plant and virus components. Can J Bot. 2005;83: 1529–1549.

[19] Romero-Brey I, Bartenschlager R. Membranous replication factories induced by plus-strand RNA viruses. Viruses. 2014;6: 2826–2857. 10.3390/v6072826 25054883 PMC4113795

[20] Le Gall O, Christian P, Fauquet CM, King AM, Knowles NJ, Nakashima N, et al Picornavirales, a proposed order of positive-sense single-stranded RNA viruses with a pseudo-T = 3 virion architecture. Arch Virol. 2008;153: 715–727. 10.1007/s00705-008-0041-x 18293057

[21] Krausslich HG, Nicklin MJ, Lee CK, Wimmer E. Polyprotein processing in picornavirus replication. Biochimie. 1988;70: 119–130. 2840974 10.1016/0300-9084(88)90166-6

[22] Sanfacon H, Wellink J, Le Gall O, Karasev A, van der Vlugt R, Wetzel T. Secoviridae: a proposed family of plant viruses within the order Picornavirales that combines the families Sequiviridae and Comoviridae, the unassigned genera Cheravirus and Sadwavirus, and the proposed genus Torradovirus. Arch Virol. 2009;154: 899–907. 10.1007/s00705-009-0367-z 19350366

[23] Sanfacon H, Zhang G, Chisholm J, Jafarpour B, Jovel J. Molecular biology of Tomato ringspot nepovirus, a pathogen of ornamentals, small fruits and fruit trees In: Teixeira da Silva J, editor. Floriculture, Ornamental and Plant Biotechnology: Advances and Topical Issues (1st Edition). London, UK: Global Science Books; 2006 pp. 540–546.

[24] Wang A, Han S, Sanfacon H. Topogenesis in membranes of the NTB-VPg protein of Tomato ringspot nepovirus: definition of the C-terminal transmembrane domain. J Gen Virol. 2004;85: 535–545. 14769910 10.1099/vir.0.19612-0

[25] Zhang SC, Zhang G, Yang L, Chisholm J, Sanfacon H. Evidence that insertion of Tomato ringspot nepovirus NTB-VPg protein in endoplasmic reticulum membranes is directed by two domains: a C-terminal transmembrane helix and an N-terminal amphipathic helix. J Virol. 2005;79: 11752–11765. 16140753 10.1128/JVI.79.18.11752-11765.2005 PMC1212610

[26] Zhang G, Sanfacon H. Characterization of membrane-association domains within the Tomato ringspot nepovirus X2 protein, an endoplasmic reticulum-targeted polytopic membrane protein. J Virol. 2006;80: 10847–10857. 16928745 10.1128/JVI.00789-06 PMC1641798

[27] Han S, Sanfacon H. Tomato ringspot virus proteins containing the nucleoside triphosphate binding domain are transmembrane proteins that associate with the endoplasmic reticulum and cofractionate with replication complexes. J Virol. 2003;77: 523–534. 12477857 10.1128/JVI.77.1.523-534.2003 PMC140641

[28] Sanfacon H. Investigating the role of viral integral membrane proteins in promoting the assembly of nepovirus and comovirus replication factories. Front Plant Sci. 2013;3: 313 10.3389/fpls.2012.00313 23439982 PMC3557413

[29] Walker M, Chisholm J, Wei T, Ghoshal B, Saeed H, Rott M, et al Complete genome sequence of three tomato ringspot virus isolates: evidence for reassortment and recombination. Arch Virol. 2015;160: 543–547. 10.1007/s00705-014-2240-y 25267178

[30] Rott ME, Gilchrist A, Lee L, Rochon D. Nucleotide sequence of tomato ringspot virus RNA1. J Gen Virol. 1995;76: 465–473. 7844569 10.1099/0022-1317-76-2-465

[31] Petersen TN, Brunak S, von Heijne G, Nielsen H. SignalP 4.0: discriminating signal peptides from transmembrane regions. Nat Methods. 2011;8: 785–786. 10.1038/nmeth.1701 21959131

[32] King RD, Saqi M, Sayle R, Sternberg MJ. DSC: public domain protein secondary structure predication. Comput Appl Biosci. 1997;13: 473–474. 9283763 10.1093/bioinformatics/13.4.473

[33] Guermeur Y, Geourjon C, Gallinari P, Deleage G. Improved performance in protein secondary structure prediction by inhomogeneous score combination. Bioinformatics. 1999;15: 413–421. 10366661 10.1093/bioinformatics/15.5.413

[34] Rost B. PHD: predicting one-dimensional protein structure by profile-based neural networks. Methods Enzymol. 1996;266: 525–539. 8743704 10.1016/s0076-6879(96)66033-9

[35] von Heijne G. The signal peptide. J Membr Biol. 1990;115: 195–201. 2197415 10.1007/BF01868635

[36] Nilsson I, Johnson AE, von Heijne G. Cleavage of a tail-anchored protein by signal peptidase. FEBS Lett. 2002;516: 106–108. 11959113 10.1016/s0014-5793(02)02511-5

[37] Nothwehr SF, Folz RJ, Gordon JI. Uncoupling of co-translational translocation from signal peptidase processing in a mutant rat preapolipoprotein-A-IV with a deletion that includes the COOH-terminal region of its signal peptide. J Biol Chem. 1989;264: 4642–4647. 2647742

[38] Yeagle PL, Bennett M, Lemaitre V, Watts A. Transmembrane helices of membrane proteins may flex to satisfy hydrophobic mismatch. Biochim Biophys Acta. 2007;1768: 530–537. 17223071 10.1016/j.bbamem.2006.11.018

[39] Chisholm J, Zhang G, Wang A, Sanfacon H. Peripheral association of a polyprotein precursor form of the RNA-dependent RNA polymerase of Tomato ringspot virus with the membrane-bound viral replication complex. Virology. 2007;368: 133–144. 17658576 10.1016/j.virol.2007.06.032

