# Supplementary figures and images for: Characterization of a Non-Canonical Signal Peptidase Cleavage Site in a Replication Protein from Tomato Ringspot Virus
Source: PLoS One. 2016 Sep 2;11(9):e0162223. doi: 10.1371/journal.pone.0162223 (PMC5010249; doi:10.1371/journal.pone.0162223)

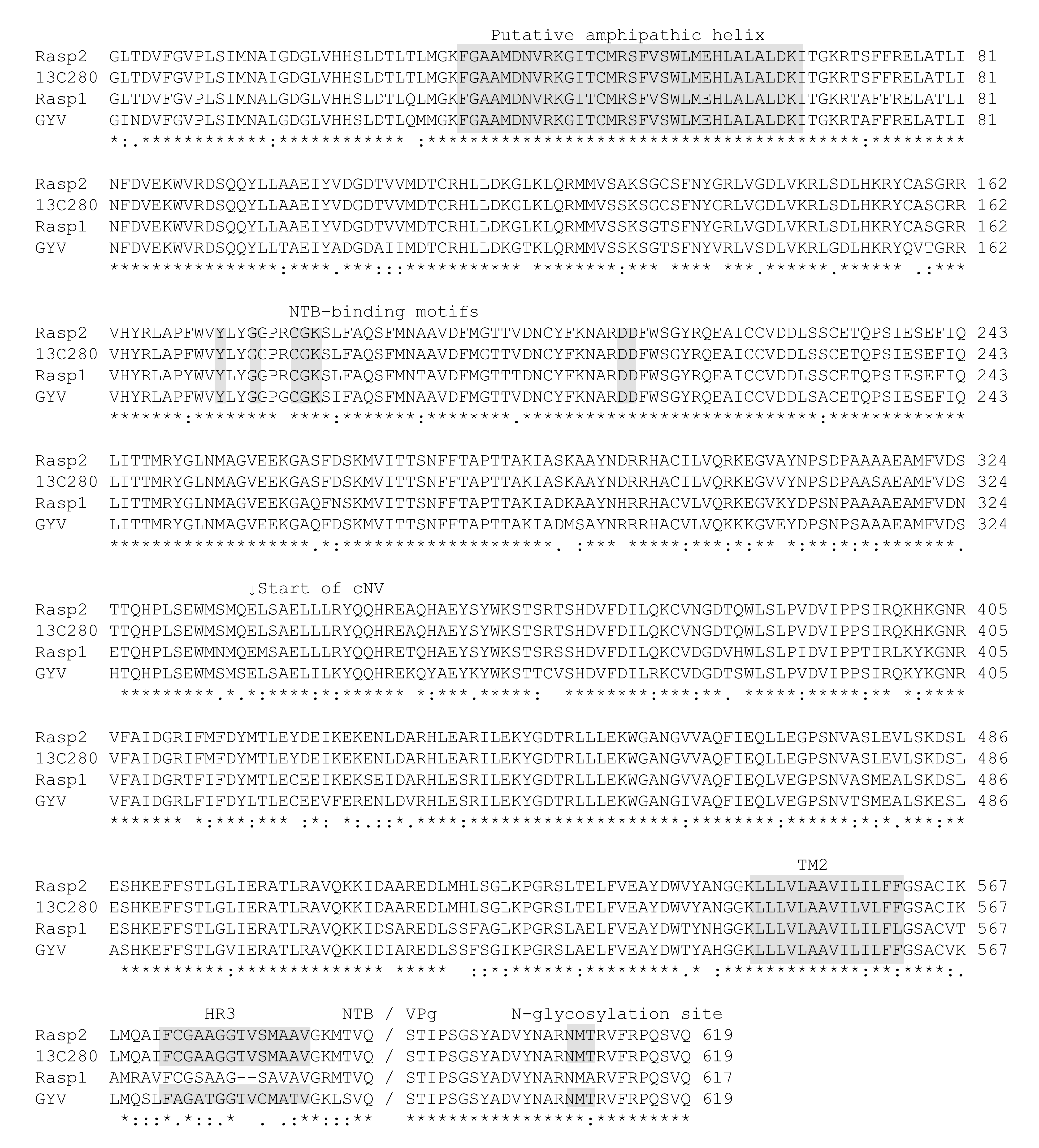

Supplement: S1 Fig — Starting amino acid for the cNV truncated protein is indicated with an arrow. Previously identified motifs are highlighted with the grey boxes and defined above the alignment and in the text. The border between the NTB and VPg domain (NTB/VPg) is also shown. As defined in ClustalW2, an asterisk (*) indicates conserved residues, a colon (:) indicates residues with strongly similar properties and a period (.) indicates residues with weakly similar properties. (TIF) [file pone.0162223.s001.tif]
